# A Rapid, Self-confirming Assay for HIV: Simultaneous Detection of Anti-HIV Antibodies and Viral RNA

**DOI:** 10.4172/2155-6113.1000540

**Published:** 2016-01-31

**Authors:** Zongyuan Chen, Hui Zhu, Daniel Malamud, Cheryl Barber, Yhombi Yvon Serge Ongagna, Rubina Yasmin, Sayli Modak, Malvin N. Janal, William R. Abrams, Richard A. Montagna

**Affiliations:** 1 Rheonix, Inc., Ithaca, New York, USA; 2 New York University College of Dentistry, Department of Basic Sciences, New York, USA; 3 New York University School of Medicine, Department of Medicine, New York, USA

**Keywords:** HIV diagnostics, AIDS, Loop Mediated Isothermal Amplification (LAMP), Molecular diagnostics, Rapid diagnostics

## Abstract

**Objective:**

We developed a microfluidic system to simultaneously detect host anti-HIV antibodies and viral RNA in the same specimen in order to satisfy two important diagnostic criteria, especially within resource-limited settings. First, the system can detect acute HIV infection and allow immediate confirmation of a seropositive screening result by detection of HIV RNA. It also addresses the well-known "seroconversion window" during early HIV infection when antibodies are not yet detectable and viral loads are at their highest.

**Methods:**

We first developed and optimized two separate manual assays for the detection of host anti-HIV antibodies and viral RNA and then converted them to the microfluidic system. We optimized a commercially available serologic assay to run within the microfluidic device while we incorporated the isothermal LAMP assay to detect the presence of viral RNA. The microfluidic device and instrumentation were developed to simultaneously perform both assays without any user intervention.

**Results:**

The finalized system consists of a disposable injection molded and film-laminated microfluidic CARD disposable device and a portable, software controlled instrument, which together can automatically perform all steps of both assays without any user intervention after the initial loading of samples and reagents. The microfluidic CARD cartridge has multiple microchannels, valves, pumps and reservoirs, which perform the immunoassay, isolates viral RNA for detection by magnetic bead based purification, and Reverse Transcriptase loop-mediated isothermal amplification (RT-LAMP). The microfluidic system was able to detect host anti-HIV antibodies and viral RNA in either a blood or saliva sample.

**Conclusion:**

The ability to detect antibodies and simultaneously confirm a seropositive HIV-RNA result provides healthcare workers with a complete and accurate appraisal of a patient's infection status in the earliest stages of the disease and represents an important tool for the "Test and Treat" and "Treatment as Prevention" approaches for controlling the HIV epidemic.

## Introduction

The global HIV/AIDS epidemic continues to be fueled by individuals who do not know they are infected and thus they continue to transmit the virus. The most effective means to control the spread of HIV is via early diagnosis [1–5], education, and behavioral modification [3,4,6–8]. However, unlike developed countries, low resource settings often lack sophisticated equipment, trained personnel, and facilities required to effectively test clinical specimens. In addition, since patients may have to travel to distant centers, loss to follow up also becomes a concern, particularly when repeat testing is required. Historically the challenges faced in low resource settings have led to the use of rapid, easy-to-use dipstick tests to screen individuals for the presence of anti-HIV antibodies. As this class of diagnostics lacks the sensitivity and specificity of more sophisticated assays, initial seropositive results are often confirmed only by use of another manufacturer’s rapid screening test. Since the second rapid test is prone to the same analytical shortcomings of low specificity and sensitivity as the first, more sensitive confirmatory approaches are warranted. To meet the needs of resource-limited settings, an assay must be rapid, sensitive, specific, easy-to-perform and inexpensive. Ideally, the assay should be run on an integrated diagnostic platform that can process samples from the subject through to analytical result. Such a system will facilitate the “Test and Treat” approach to controlling the HIV epidemic [4,6,8,9].

The U.S. Centers for Disease Control and Prevention estimates that the interval between HIV infection and the appearance of HIV-1 RNA is approximately 7–10 days [10]. Therefore, HIV RNA based detection will not only confirm initial rapid immunoassay results, but also facilitate early detection of HIV infection before the development of anti HIV antibodies. Recently, considerable efforts have focused on developing portable devices for detection of HIV RNA in resource-limited settings. Although PCR has been widely utilized in microfluidic systems to amplify DNA or RNA, isothermal amplification methods have attracted increasing attention due to their simplicity and sensitivity [11]. Among commercially available isothermal systems, Loop-mediated isothermal AMPlification (LAMP) stands out for its rapidity, sensitivity and ability to amplify both DNA and RNA. Sun et al. [12] used a SlipChip microfluidic device to evaluate the performance of digital reverse transcription- (dRT-LAMP) for quantification of HIV viral RNA. Jangam et al. [13] demonstrated a proof-of-concept platform for detection of HIV-1 in blood with a battery-operated portable analyzer. Wang et al. [14] reported on detection of HIV-1 DNA on an integrated microfluidic device including cell lysis, extraction of DNA, polymerase chain reaction (PCR), and optical detection. Finally, Liu et al. [15] described a simple-to-use, low-cost, pump-free, sedimentation-assisted plasma separator and demonstrated RNA extraction and LAMP based detection of HIV-1 RNA on the same microfluidic device utilizing a silica membrane.

We have previously reported the simultaneous detection of antibodies and viral RNA in both blood and saliva using a portable device equipped with an open architecture dual path microfluidic cartridge [16]. To improve its sensitivity, reliability and throughput, we describe further improvements to the system: replacing silica-based nucleic acid purification with a magnetic bead-based process; replacing PCR with LAMP; and increasing the number of samples analyzed from one to four on a single CARD microfluidic device. In order to develop the fully automated system to perform both the serological and molecular assays, the corresponding benchtop assays were first optimized and then adapted to the automated Rheonix CARD assay. Samples of either plasma or saliva are introduced into the disposable Rheonix CARD cartridge and under the control of the instrument’s software, specimens are directed into two microfluidic pathways. One pathway is used to analyze specimens for the presence of anti-HIV antibodies while the other pathway isolates viral RNA using magnetic bead capture technology and RT-LAMP to amplify the viral target. The resulting dual assay provides immediate molecular confirmation of the serological results and also serves to decrease the time from infection to detection, thus shortening the “seroconversion window.”

## Materials and Methods

### Plasma and saliva samples

Whole Mouth Stimulated Saliva (WMSS) and blood were collected as previously described [17] under an approved IRB protocol: NYU IRB #05-51.

### Virus

HIV-1 MN virus was obtained from Advanced Biotechnologies, Inc. (Columbia, MD).

HIV-1/2 Antibody Lateral Flow Assay (LFA) strips *and* HIV Antibody (positive control) were generous gifts provided by OraSure Technologies, Inc. (Bethlehem, PA).

### Benchtop antibody detection

Manual detection of anti-HIV antibodies using the OraSure lateral flow assay (LFA) test strips was performed by mixing 45 µl of high salt lateral flow (HSLF) buffer, composed of 270 mM NaCl, 1% (w/v) BSA (Sigma Aldrich, Cat. Number A-2153), 0.5 % (v/v) Tween-20 in 100 mM HEPES, pH 7.4) with 20 µl of sample, and then allowing the mixture to flow up the lateral flow test strip. Two separate aliquots of 45 µl of HSLF buffer were then allowed to flow up the test strip to wash away any unbound materials.

### Benchtop RNA isolation

Viral RNA was isolated from samples using the Dynabeads SILANE viral NA kit (Invitrogen/Life Technologies AS, Oslo, Norway) employing the manufacturer’s recommended procedure. Briefly, 50 µl of Proteinase K (Sigma, P4850, 14 mg/ml) was first mixed with 200 µl of sample followed by mixing and incubation with 300 µl of lysis/binding buffer for 5 min at room temperature. 150 µl isopropyl alcohol (IPA) and 50 µl of Dynabeads were added to the mixture and incubated for 5 min on a roller. After capture of beads on a magnetic rack, 850 µl of Wash Buffer 1 were used to wash the beads two times. Then, these washes were repeated with Wash Buffer 2. After aspirating the wash buffer, the tube was left to air dry for 10 minutes to remove any residual alcohol and then RNA eluted with 50 µL of Elution Buffer.

### Benchtop LAMP assay

Analytical sensitivity of the RT-LAMP assays was determined with a dilution series of HIV-1 MN RNA isolated as described above. Master mix (OptiGene ISO-001) was combined with 0.2 Units of avian myeloblastosis virus reverse transcriptase (AMV-RT) and 3 pairs of primers [18] targeting the HIV-1 p24 gene (Table 1) in a single tube with a final volume of 25 µl including 3–4 µl RNA at 65 °C. SYBR Green I interchelating dye, included in the OptiGene Mastermix formulation and allows following real-time fluorescence in a Genie III (OptiGene, Horsham, UK) portable isothermal amplification device. In a manner similar to melting curve analyses, annealing curves were obtained immediately following LAMP to allow post- amplification display of products by increasing the temperature to 92 °C and gradually cooling to 85°C.

### Microfluidic CARD assay

**Figure 1:**
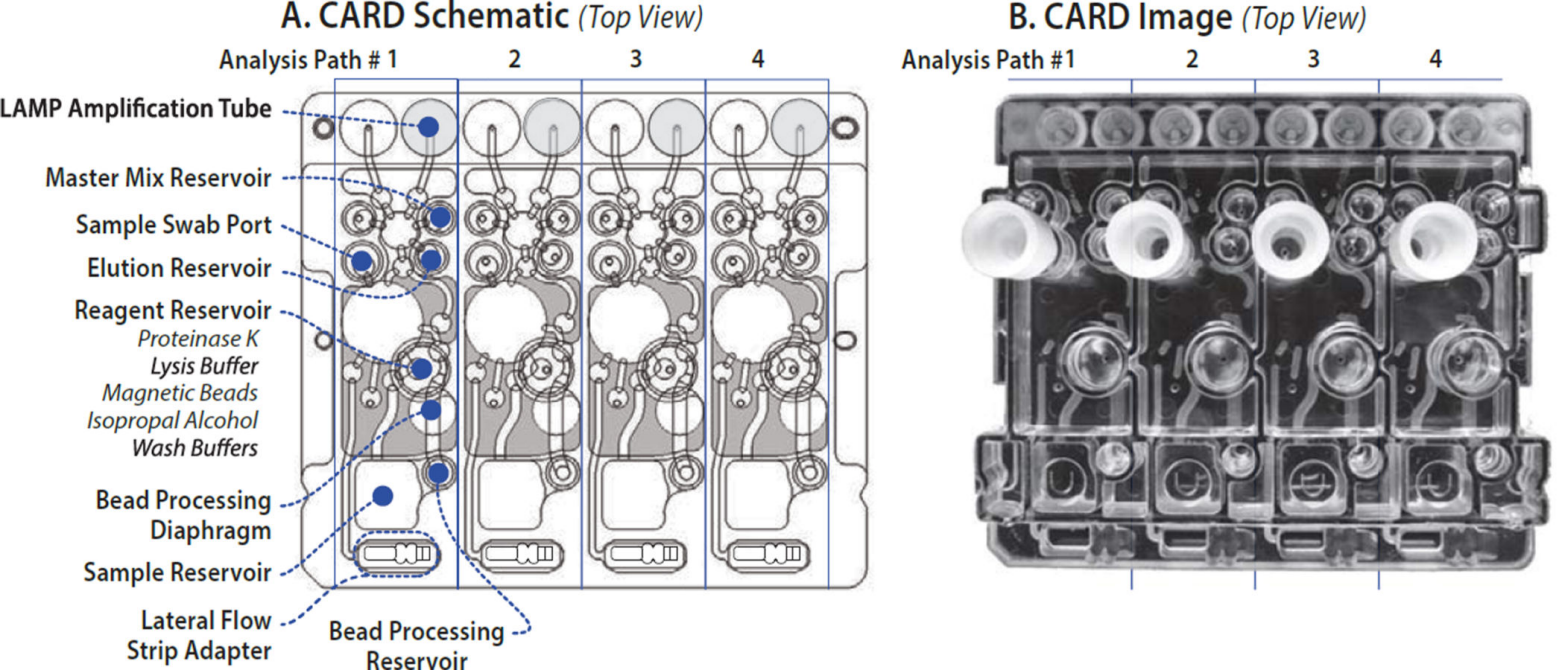
Schematic and images of Dual Path CARD cartridge for screening and confirmatory analysis of HIV infection. (A) Top view of schematic indicating the positions of the sample swab input ports, the types of reagent wells, and lateral flow strip adapters. (B) Top view image of the injection molded CARD cartridge. Flow of sample is initiated once the sample is introduced in the Sample Reservoir. Twenty µl of the sample is pumped through the various diaphragm pumps and channels to the Lateral Flow Strip Adapter compartment where the lateral flow antibody assay occurs. Simultaneously, the remaining 200 µl of the sample reacts with listed reagents which are loaded to Reagent Reservoir and pumped to Sample Reservoir or Bead Processing Reservoir. Beads are captured at Bead Processing Diaphragm and eluent is pumped to Elution Reservoir. Eluent and LAMP master mix from Master Mix Reservoir are pumped to LAMP Amplification Tube for LAMP reaction.

The antibody and viral RNA assays were simultaneously performed on the CARD cartridge (Figure 1) by first loading 220 µl of sample to the Sample Reservoir. Anti-HIV antibodies were detected using the same LFA test strips used in benchtop assays by inserting the strips into the CARD cartridge’s Lateral Flow Strip Adaptor. Antibodies were detected by pumping a 20 µl aliquot from the Sample Reservoir to the Retainer Reservoir. HSLF buffer (140 µl) was loaded into the Reagent Reservoir and 45 µl pumped to the Retainer Reservoir where it was mixed with the sample. The mixture was then pumped to the Lateral Flow Strip Adaptor where it flowed up the LFA test strip. After two minutes, two additional aliquots of 45 µl of HSLF buffer were automatically transferred to the Lateral Flow Strip Adaptor to wash unbound protein and reporter gold onto the waste pad.

In parallel with the antibody detection process, viral RNA was also simultaneously isolated by following a modified protocol for processing on the CARD cartridge. Reagents including Proteinase K, lysis/binding buffer, bead-isopropyl alcohol (IPA) mixture, wash buffer-1 and -2 that had previously been preloaded to the Reagent Reservoir were pumped to the Sample Reservoir or Bead Processing Reservoir, as required. 50 µl of Proteinase K (14 mg/ml) was pumped to the sample reservoir and mixed with the remaining 200 µl sample and incubated for 2 min at room temperature. Then 300 µl of lysis/binding buffer was pumped into the sample reservoir, mixed and incubated for 5 min at room temperature. Finally, 10 µl of Dynal magnetic beads premixed with 150 µl of IPA were added to the lysate and incubated for 5 min to allow binding of nucleic acids to the beads. The resulting mixture was then pumped to the Bead Processing Reservoir where permanent magnets were pneumatically moved into position to capture the beads. The captured beads were then washed with 100 µl of Wash Buffer 1 followed by two additional washes with 70 µl of Wash Buffer 1. The same wash steps were repeated with Wash buffer 2. After pumping air for 5 minutes to dry the beads, 50 µl of elution buffer was introduced to the Elution Reservoir and pumped to the Bead Processing Reservoir to elute the nucleic acids from the beads. LAMP Master mix (24 µl) from the MM1 reservoir, along with 1.5 µl of isolated RNA from the Elution Reservoir were pumped to the nucleic acid amplification tubes where RT- LAMP was carried out by heating to 65 °C for 22 min while the instrument acquired real-time fluorescence signals at 1 min intervals. After the RT-LAMP reaction was completed, an annealing curve was obtained by heating the amplicons to 92 °C and cooling to 85 °C with fluorescence signals obtained at 12 second intervals.

### RNA quantification

RNA was quantified spectrophotometrically for both benchtop and CARD cartridge methods. The Nanodrop (ThermoFisher) was used to determine concentrations down to approximately 400 ng/ml while more dilute samples in the range of 2.5 to 50 ng/ml were analyzed using the Quant-iT RiboGreen RNA Assay Kit (Life Technologies, Grand Island, NY).

### Statistical analysis

Virus detection data were summarized as mean +/− SD for triplicate assays on the benchtop (BT) and CARD cartridge assays. Sensitivity and precision were assessed by comparing means and coefficients of variation for manual and automated CARD cartridge results, respectively.

## Results

To develop a fully automated assay for the simultaneous detection of anti-HIV antibodies and HIV RNA in blood and saliva samples, we first separately optimized procedures for the two assays on the benchtop and then adapted those processes for the microfluidic CARD device. In parallel a software-controlled instrument into which the CARD device can be mounted was developed so that unattended analysis could be achieved.

### Benchtop HIV RNA extraction and RT-LAMP detection

**Figure 2:**
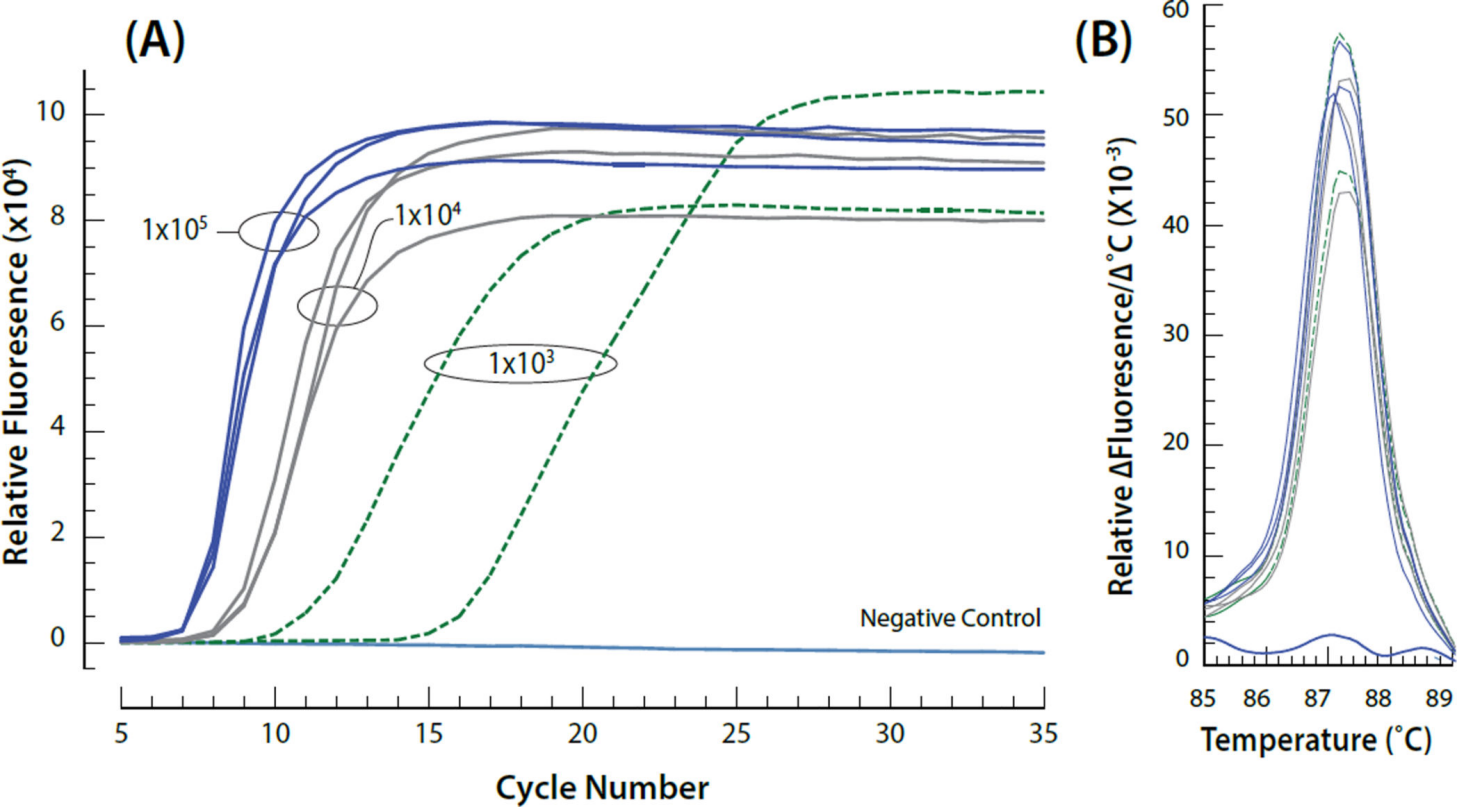
(A) Examples of on-CARD real time RT-LAMP amplification as a function of different input viral concentrations. The viral particles/mL are indicated on the graph for each group of curves. Viral nucleic acid (RNA) was isolated and the resulting template amplified using LAMP entirely on the CARD cartridge (B) Annealing curves for the amplified target resulting from the different viral concentrations.

A workflow was developed that first analyzed a specimen for the presence of anti-HIV antibodies and then introduced chaotropic agents required to lyse the virions to release viral RNA. Starting with a 220 µl aliquot of the test specimen, 20 µl was first evaluated for anti-HIV antibodies using commercially available lateral flow test strips. The remaining 200 µl was then processed using the Dynabeads SILANE viral NA kit as described in Materials and Methods. Benchtop RNA extraction and LAMP analysis of HIV-spiked saliva samples indicated that viral RNA could be reproducibly detected at concentrations as low as 10^3^ viral particles/ml of the original blood or saliva sample (Figure 2).

### Automatic Performance of Immunoassay and RT-LAMP Detection of HIV on CARD

**Figure 3:**
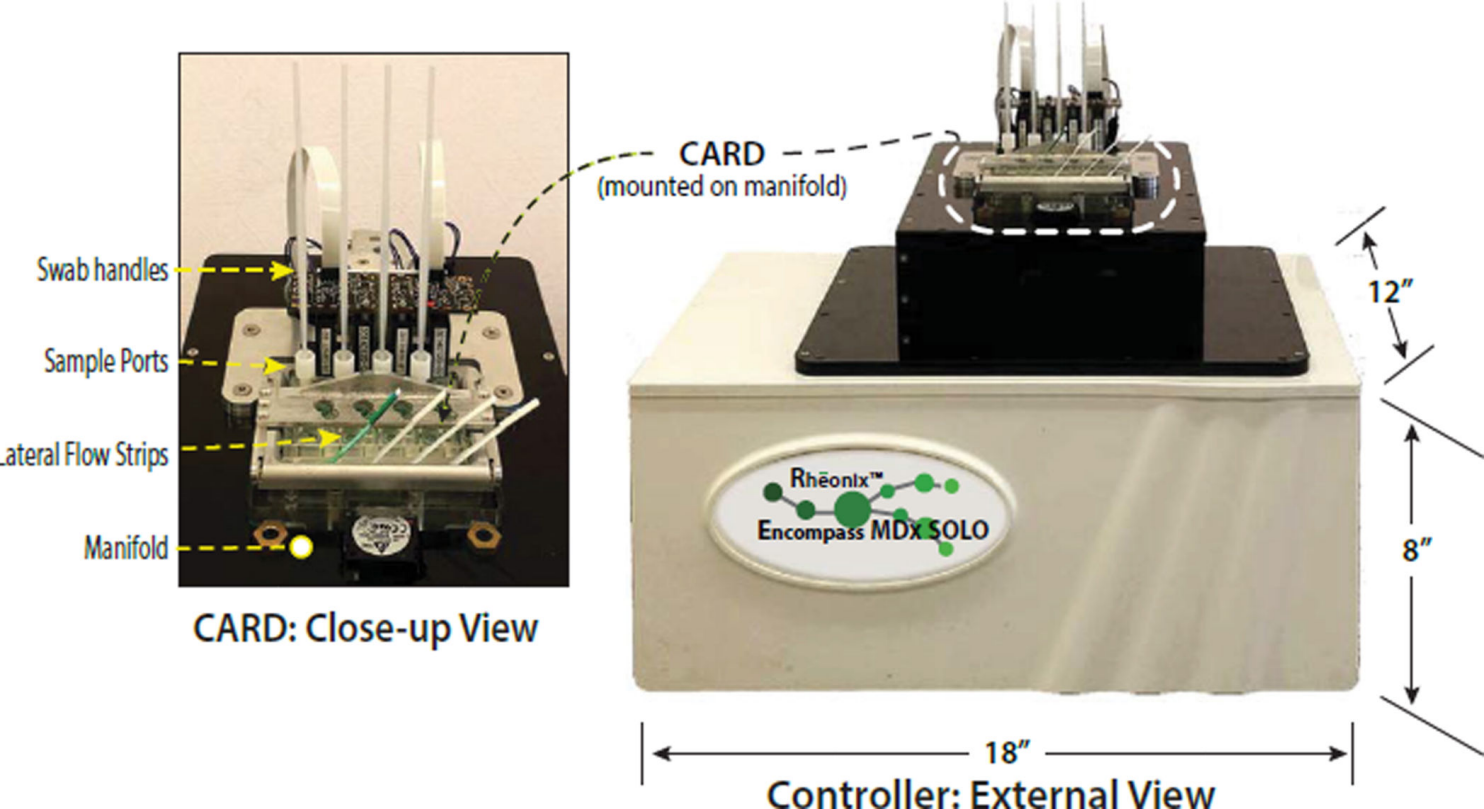
Image of the Encompass SOLO CARD processor with a close-up of a CARD cartridge mounted on the processor. The positions of the sample ports and lateral flow strips are indicated.

The assay parameters developed for manual detection of anti-HIV antibodies using lateral flow technology and viral RNA by LAMP were adapted for the CARD cartridge. A plastic injection molded and film laminated disposable microfluidic device was designed and fabricated to automatically complete all sample preparation and target detection simultaneously under processor control without user intervention. To minimize the cost per test, the dual pathway CARD cartridge was designed to simultaneously process four separate specimens (Figure 1). The disposable injection molded and film-laminated microfluidic CARD cartridge has multiple microchannels, valves, pumps and reservoirs. A portable processor was also designed and fabricated to control all immunoassay and RT-LAMP assay steps on the CARD cartridge (Figure 3). All CARD assay functions are under software control, including flow and mixing rates of buffers, antibody assay functions and RT-LAMP with real-time data display and acquisition of the fluorescence signals. Using the Reonix Encompass SOLO instrument, viral RNA was recovered and detected via RT-LAMP on the CARD cartridge using the modified protocol described in Materials and Methods. Antibody detection on the CARD cartridge was achieved in parallel with viral RNA detection in both blood and saliva samples.

### Preparation of clinical samples for CARD assay

**Figure 4:**
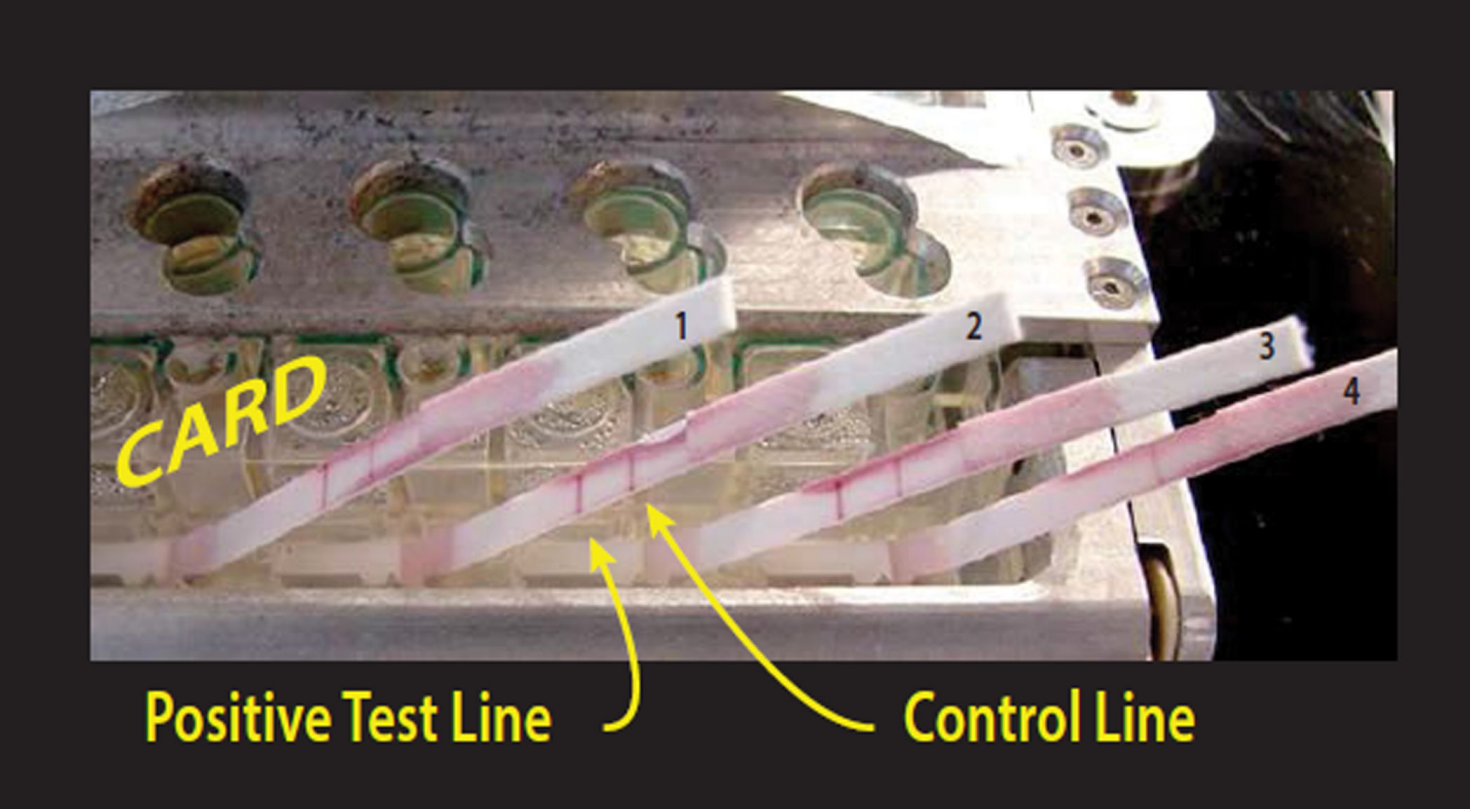
Image of developing lateral flow strips for detection of the presence of HIV antibody after approximately 10 min from the initiation of processing. Full development of the immunochromatogram requires approximately 20 min.

**Figure 5:**
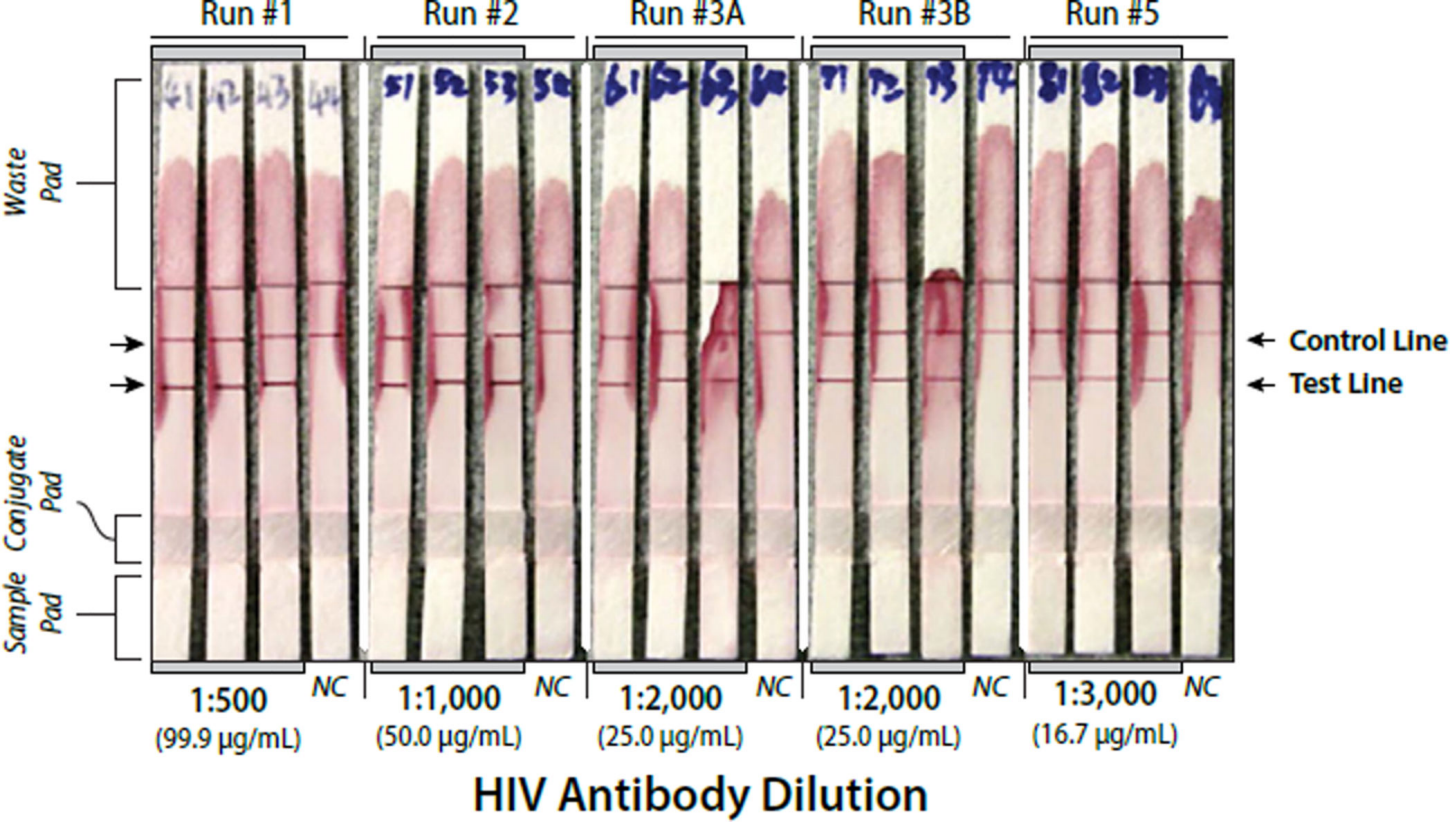
Lateral flow strips showing detection of four different HIV antibody concentrations spiked into the saliva sample. Run numbers 3A and 3B are duplicate analyses. NC = Negative Control. The protein concentrations for each antibody dilution are indicated on the graph.

**Figure 6:**
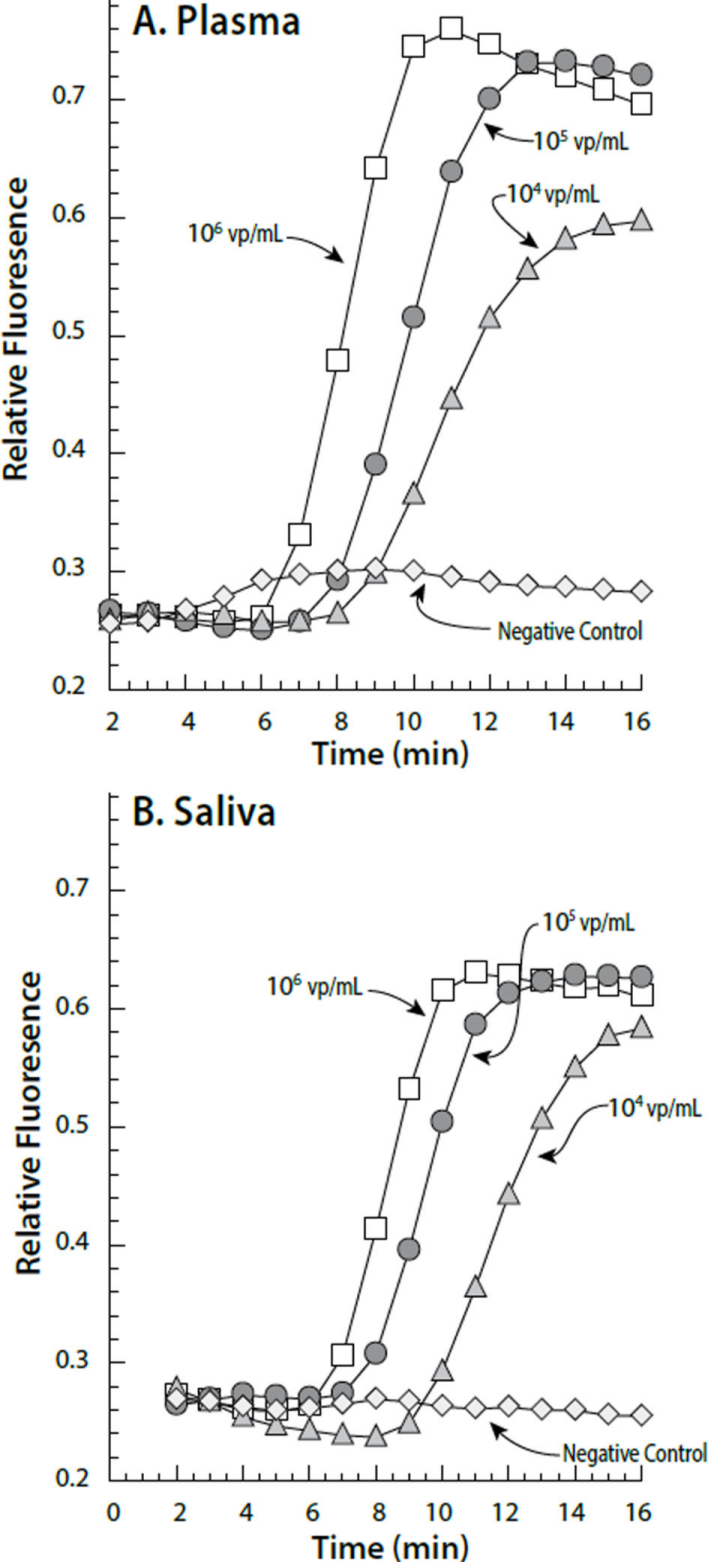
Fluorescence response observed from CARD based LAMP amplification of HIV virus particles spiked into either Plasma (A) or Saliva (B). The Negative Controls were either plasma or saliva without added HIV.

**Figure 7:**
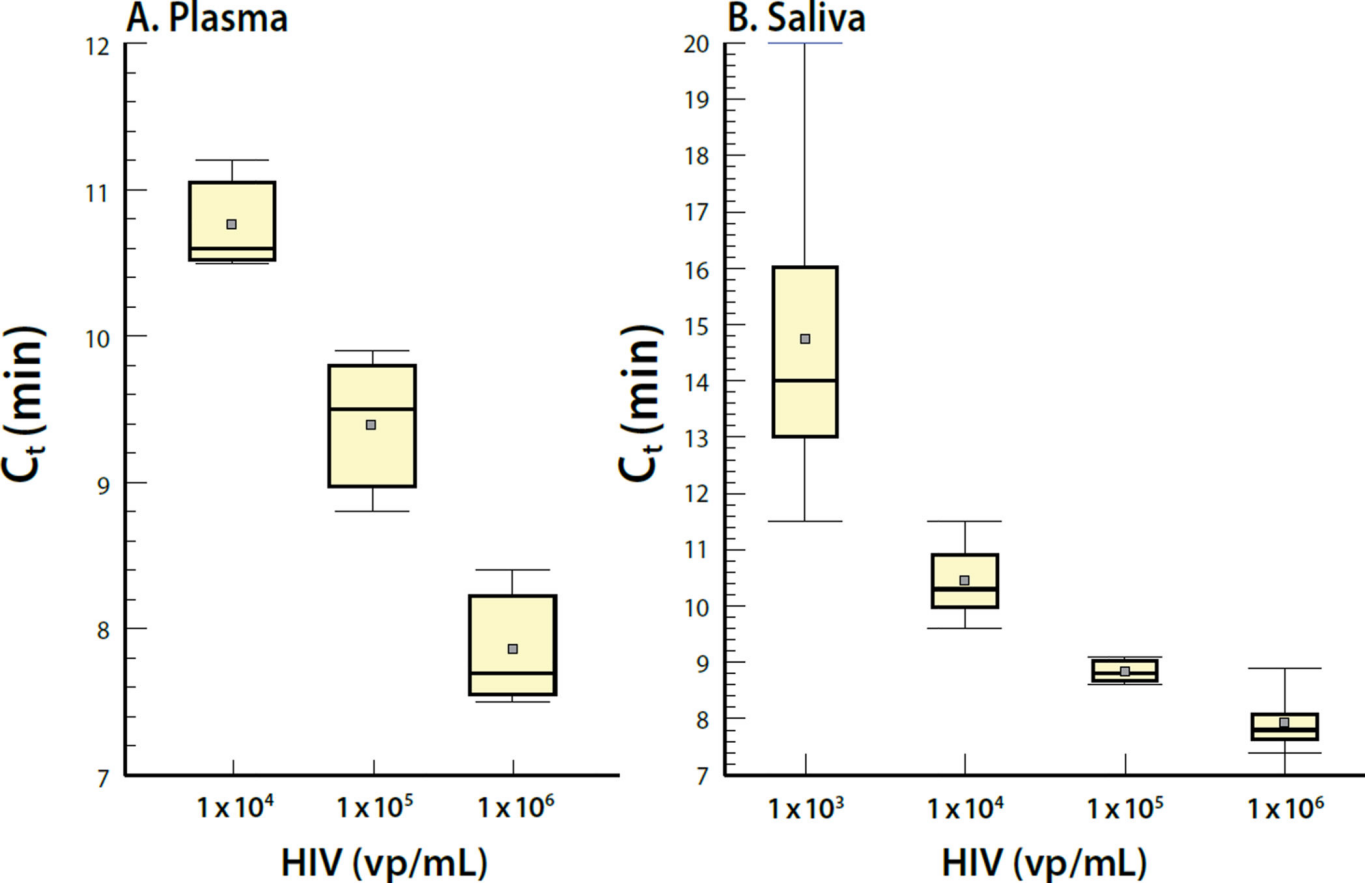
CARD detection of HIV nucleic acid in plasma (A) or saliva (B). Plasma and saliva samples were analyzed in triplicate at each virus concentration. Means are indicated by the gray squares. Statistical analysis of the LAMP results reveals that the median CV values ranged from 7 to 43.5%.

For development of the CARD assays at Rheonix facilities, high concentrations of HIV-1 MN virus were first inactivated/lysed at the NYU BSL 2 using Proteinase K and the Dynabeads SILANE viral NA lysis buffer, as described in Materials and Methods. Rheonix personnel then used this material at their facilities to spike WMSS samples for testing on CARD cartridges. Since this study was designed to detect anti-HIV antibodies and viral RNA in saliva as well as blood, HIV antibody was spiked in WMSS at dilutions ranging from 1:500 to 1:3000. The automated CARD cartridge process started with 200 µl of saliva sample added to the sample reservoirs on the CARD cartridge and then 20 µl of each were immediately processed through one microchannel pathway to detect anti-HIV antibodies using lateral flow test strips impregnated with anti-IgG antibodies (control line) and HIV viral glycoproteins (test line). The lateral flow assay took approximately 15 min to complete, with the previously confirmed seropositive clinical specimens yielding clearly discernible signals at the test and control lines and the seronegative specimens yielding a signal at only the control line (Figure 4). An additional 20 saliva specimens (15 of which were spiked with a range of antibody dilutions and 5 negative controls) were evaluated on a total of five separate CARD cartridges. All 15 seropositive specimens yielded positive lateral flow results while all 5 negative specimens tested negative (Figure 5). Analysis of the same saliva specimens on the CARD cartridge for the presence of viral RNA was accomplished by processing the remaining 180 µl of the original specimens while the antibody assay was simultaneously underway. To mimic the procedures that would be used in the field and to avoid a “double” lysis, the remaining 180 µl of the original specimens was lysed prior to the addition of 20 µl of pre-lysed HIV-1 MN virus (10^8^ virus particles/mL). As described in Materials and Methods, the lysate was mixed with the IPA-bead mixture for RNA binding, washing, drying and elution. Isolation of viral RNA on the CARD cartridge took approximately 56 minutes while the detection by RT-LAMP required an additional 20 minutes. The generation of the annealing curve required 4 additional minutes, resulting in a total of 80 minutes to obtain both antibody and viral RNA from the samples. The RT-LAMP results obtained from analysis of both saliva and plasma specimens spiked with clinically relevant concentrations of virus were similar (Figure 6), with analytical sensitivities in the range of 10^3^ viral particles per milliliter (Figure 7). Furthermore, analysis using benchtop RT-LAMP and on the CARD cartridge showed concordance of input virus and resulting C_t_ values, supporting the ability of the CARD cartridge to perform similarly to the more labor intensive benchtop processes (Table 2). We aimed to achieve an upper limit of 20% variation for the nucleic acid assay confidence limits, and that was achieved in all but the lowest concentration (10^3^ viral particles/ mL) of virus in saliva. The CV ranged from from 2 to 7%, but rose to 22% at the lowest virus concentration (10^3^) in saliva. There was close agreement in the mean absolute levels of Ct in plasma and saliva in the range of 10^4^ to 10^6^ levels typically seen in subjects. Variability was also similar in the two fluids, where all CVs were less than 10% (Figure 7). All samples showed acceptable reproducibility, with CVs of less than 20%, although the CARD cartridge was slightly less sensitive and precise than the corresponding bench top process for 10^3^ vp/mL samples.

Proof-of-concept Results of Direct Application of Swab to CARD cartridge and Safe and Easy Handling of Raw Samples by Finding an Appropriate Storage/lysis Buffer

### Direct application of sample to the CARD cartridge

While the present work utilized the introduction of liquid specimens into the CARD cartridge, preliminary experiments were performed to determine the feasibility of directly introducing swabs with adsorbed WMSS or finger stick blood samples to the CARD cartridge. Several commercially available swabs designed to collect both saliva and finger stick blood samples were evaluated. Since the titer of virus and antibodies is generally lower in saliva than in blood [19,20] the swabs were evaluated for their ability to adsorb virus and protein from known samples as well as the ease of release for subsequent analysis. Swabs from Copan Diagnostics (Murrieta, CA) and Puritan Medical (Guilford, ME) were compared and the Copan mini-swabs (Table 3) were selected based upon superior ability to absorb and subsequently release viral particles, providing higher yields of virus. Direct application of a swab into the CARD cartridge was tested using an adaptor specifically designed to accept a swab (Figure 1). The design of the adaptor port allows leak free flow of buffer through the swab for effective mixing and release of biomaterials. Lysis buffer is pumped directly onto the swab after it is inserted into the port, which combines wash and lysis functions and avoids dilution within the CARD cartridge that could limit detection of low titers of virus in saliva.

### Exploration of lysis buffer compatible with antibody detection for safe and easy handing of raw samples

**Figure 8:**
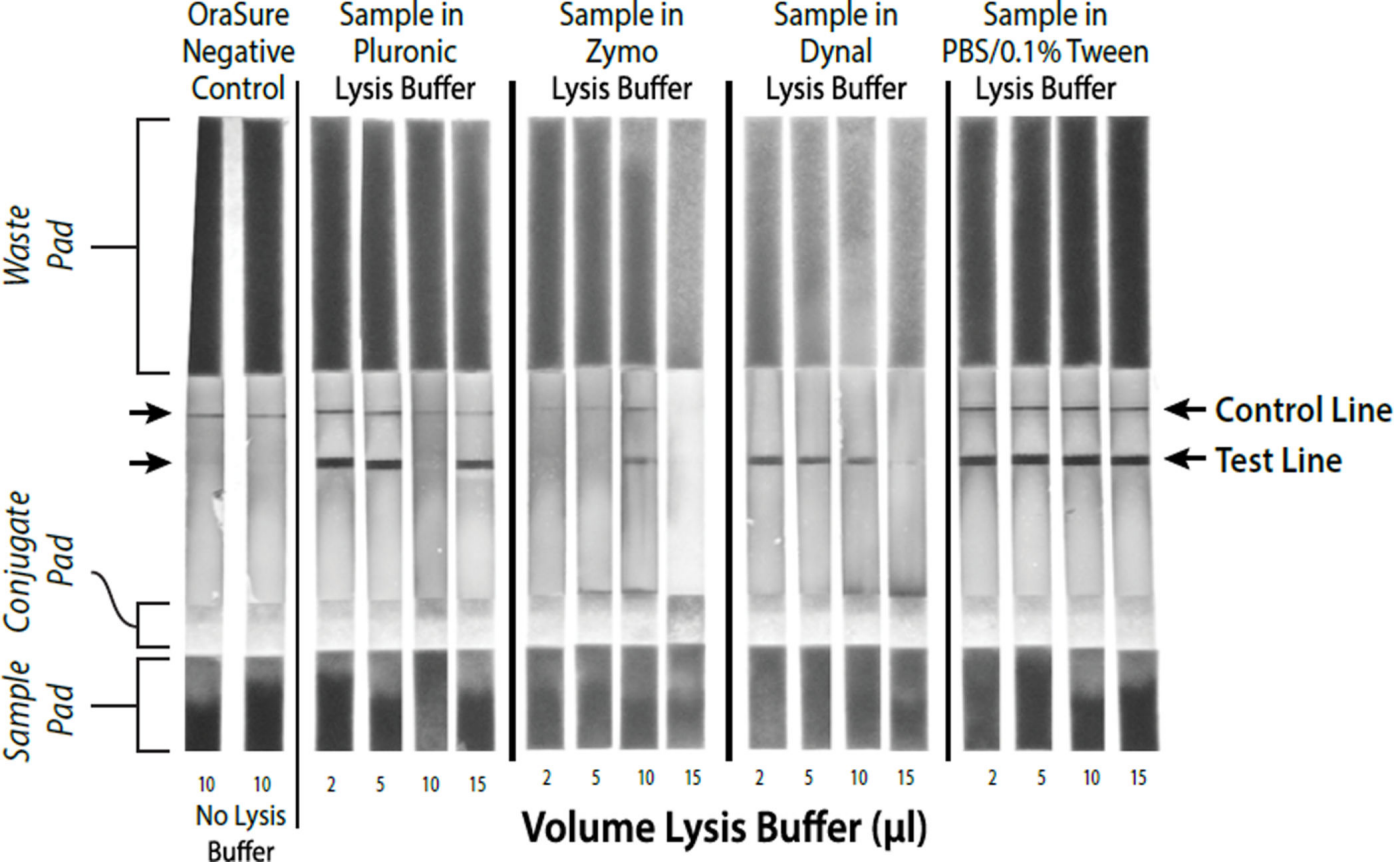
Comparison of different viral nucleic acid extraction buffers for their ability to preserve host antibody detection during benchtop processing. The 10 µl sample in Pluronic Lysis Buffer was a failed LF analysis, possibly due to operator error, but it should be noted that out of over 35 LF analyses, this is the only failed lateral flow analysis.

Simultaneous detection of antibodies and viral RNA in the same specimen poses challenges with respect to sample handling options. Generally, buffer systems that are able to maintain antibody reactivity do not effectively lyse virions to release RNA. Conversely, buffers designed to lyse virions and release viral RNA also denature proteins, thus decreasing or destroying antibody reactivity. To avoid collecting two different samples from subjects our initial efforts focused on defining a buffer system that would allow detection of both proteins and nucleic acids. Several commercial and in-house buffer systems were evaluated, including phosphate buffered saline with 0.1% Tween-20 (PBST), Zymo Research lysis buffer (Zymo Research catalog number R1035), Dynabeads Silane Viral Nucleic Acid Lysis Buffer (Invitrogen catalog number 37011D), and 15% pluronic acid F-68, 50 mM Tris-HCl, pH 6.6, 4.5 M guanidine HCL (pluronic acid buffer system). As noted in (Figure 8), both PBST and pluronic acid buffers gave strong signals for detection of host anti-HIV antibody. However, the PBST buffer system did not lyse virus efficiently and consequently low amplification was observed (data not shown). On the other hand, the pluronic acid buffer effectively lysed virions that could be amplified using LAMP. In order for the PBST buffer system to achieve LAMP results similar to those achieved using the pluronic acid buffer, it was necessary to incorporate a second lysis step with guanidine HCl to release viral RNA for LAMP detection (Figure 2). The use of a single buffer system to support both assays simplifies the device’s architecture and helps reduce cost. The pluronic acid buffer system allows the collection and lysis of a saliva sample on a swab while preserving antibody reactivity and RNA detection.

## Discussion

The serological status of infected individuals can easily be determined by use of lateral flow assays (LFAs). Due to their relative ease-of-use, such LFAs have found widespread use in resource limited settings. But, the ability to rapidly confirm the HIV status of infected individuals is critical. The dual assay described in this study provides healthcare workers with additional confidence regarding the infection status of a patient and eliminates the need for return visits for confirmatory testing. In addition, the ability to directly detect HIV virions via molecular amplification shortens the well-known “seroconversion window” during early infection. Any healthcare workers relying solely upon serological analysis would not be aware of the true HIV status of any newly infected seronegative patients. Since viral titers are highest during this period [9], the outcome of misclassifying individuals during early HIV infections has a significant impact on the subject and others in the community who might come in contact with the subject’s body fluids. Overall, the proposed dual assay format will lower the risk of transmission of infected fluids and is consistent with “Test and Treat” and “Treatment as Prevention” models [21] for infection control. The speed with which confirmed infection status can be achieved by the proposed dual assay will also reduce the time between diagnosis and initiation of appropriate therapeutic intervention. In addition, since the real time LAMP assay can be monitored viral loads can also be determined by comparing the real time fluorescence results against appropriate standards. The system is easy to use and can be operated by individuals without extensive training in resource limited settings.

While our initial work utilized RT-PCR [10] we sought to reduce the cost and complexity of the dual assay. Using the isothermal LAMP assay to detect viral RNA allowed us to reduce the complexity of the instrumentation for viral RNA while at the same time allowing a simple and simultaneous serological assay. Not including the time to prepare samples for analysis, traditional RT-PCR assays require at least 60 minutes to complete while the LAMP assay can be completed approximately 20 minutes. Factoring in the time to prepare the samples, the current CARD assay can be completed in approximately 80 minutes. As we continue our development efforts we are also striving to further reduce the total turn-around time to 20–30 minutes. Towards that end we are currently evaluating novel methods to achieve rapid viral lysis without disrupting antibody reactivity.

The ability to provide a diagnostic tool capable of detecting both anti-HIV antibodies and viral RNA does not overcome all of the challenges associated with performing such assays in resource limited settings. Towards that end, the prototype Encompass SOLO instrument was developed to provide automated control of all sample preparation and dual assay functions on the disposable microfluidic CARD cartridge. Moreover, while the results obtained in the present work utilized liquid reagents, efforts are currently underway to substitute heat-stable, lyophilized reagents that will be automatically hydrated in the instrument. Use of dried reagents will simplify the workflow required to perform the dual assay and permit more economical shipping and storage of the disposable CARD devices without cold chain handling. Since testing in resource limited settings often occurs in remote locations, the use of temperature stable reagents will also provide additional benefit to the dual assay platform.

The final system meets six of the seven ideal ASSURED criteria recommended by the World Health Organization for point-of-care testing in resource limited settings [8,9]. Those criteria include, (1) Affordable by those at risk of infection, (2) Sensitive, (3) Specific, (4) User-friendly, (5) Rapid and Robust, (6) Equipment Free, and (7) Delivered to those who need it.

Since the system developed for the simultaneous detection of both antibodies and viral RNA uses generic functions on the CARD microfluidic device, it would prove relatively straightforward to expand the CARD cartridge’s capability to perform assays for other bacterial, viral or protozoan pathogens. The availability of appropriate antigens and knowledge of target nucleic acids for emerging pathogens would allow the system’s functionality to be expanded, thus providing additional value to healthcare workers in resource limited settings.

## Figures and Tables

**Table 1:** LAMP Primers targeting HIV p24 [18].

Primer Type	Primer Sequence	Concentration (µM)
Forward #3	ATTATCAGAAGGAGCCACC	0.2
Back #3	CATCCTATTTGTTCCTGAAGG	0.2
Forward Internal	CAGCTTCCTCATTGATGGTTTCTTT TTAACACCATGCTAAACACAGT	1.6
Back Internal	TGTTGCACCAGGCCAGATAATTTT GTACTGGTAGTTCCTGCTATG	1.6
Forward Loop	TTTAACATTTGCATGGCTGCTTGAT	0.8
Back Loop	GAGATCCAAGGGGAAGTGA	0.8

**Table 2:** Bench top and CARD comparison for the ability to extract RNA using magnetic silane beads and amplify the target sequence using RT-LAMP.

Virus particles/ml	Average C_t_ (n=3)	Standard Deviation	%CV
**Bench top Extraction**			
2.5 × 10^5^	6.94	0.22	3.2
2.5 × 10^4^	8.63	0.41	4.8
2.5 × 10^3^	10.17	1.17	11.5
**CARD Extraction**			
2.5 × 10^5^	7.15	0.42	5.9
2.5 × 10^4^	8.71	0.33	3.8
2.5 × 10^3^	12.61	1.98	15.7

**Table 3:** Sweb performance.

Swab Type/ Sample #	C_t_ (min)	Annealing Temperature (°C)
**Copan M**			
#1	8.75	86.81
#2	10.0	86.70
#3	9.50	86.90
**Copan L**			
#1	12.75	87.04
#2	–	85.94
#3	10.75	86.82
**Hydroflock**			
#1	10.50	86.10
#2	12.50	85.12
#3	14.25	86.29
